# Does the use of Acellular Dermal Matrices (ADM) in women undergoing pre-pectoral implant-based breast reconstruction increase operative success versus non-use of ADM in the same setting? A systematic review

**DOI:** 10.1186/s12885-024-12978-0

**Published:** 2024-09-27

**Authors:** Hannah I. Cook, Sevasti P. Glynou, Sara Sousi, David Zargaran, Stephen Hamilton, Afshin Mosahebi

**Affiliations:** 1 Plastic Surgery Department, Royal Free Hospital, Pond Street, London, NW3 2QG UK; 2 School of Medicine, Imperial College London, London, UK; 3 Division of Surgery and Interventional Sciences, University College London, London, UK

**Keywords:** Implant-based breast reconstruction, Acellular dermal matrices, Breast cancer, Pre-pectoral breast reconstruction, Operative complications

## Abstract

**Background:**

Breast cancer is the most common malignancy among women in the UK. Reconstruction – of which implant-based breast reconstruction (IBBR) is the most common – forms a core part of surgical management of breast cancer. More recently, pre-pectoral IBBR has become common as technology and operative techniques have evolved. Many surgeons use acellular dermal matrix (ADM) in reconstruction however there is little evidence in literature that this improves surgical outcomes. This review will assess available evidence for surgical outcomes for breast reconstructions using ADM versus non-use of ADM.

**Methods:**

A database search was performed of Ovid Medline, Embase, Cochrane Central Register of Controlled Trials and Cochrane Database of Systematic Reviews (2012–2022). Studies were screened using inclusion and exclusion criteria. Risk of Bias was assessed using the Newcastle Ottawa scale and ROBIS tools. Analysis and meta-analysis were performed.

**Results:**

This review included 22 studies (3822 breast reconstructions). No significant difference between overall complications and failure rates between ADM and non-ADM use was demonstrated. Capsular contracture, wound dehiscence and implant rippling had significant differences however these results demonstrated high heterogeneity thus wider generalisation may be inaccurate. Patient quality of life scores were not recorded consistently or comparably between papers.

**Conclusions:**

This review suggests a lack of significant differences in most complications between ADM use and non-use for pre-pectoral IBBR. If no increase in complications exists between groups, this has significant implications for surgical and legislative decision-making. There is, however, inadequate evidence available on the topic and further research is required.

**Supplementary Information:**

The online version contains supplementary material available at 10.1186/s12885-024-12978-0.

## Introduction

Breast cancer is the most common malignancy diagnosed among women in the UK, accounting for 30% of female cancers in 2019 [1]. Despite a 25% increase in incidence since 1995, the overall mortality rate has fallen by 40% in the same period [1]. These changes have been, in part, attributed to the introduction of the UK breast cancer screening programme at the end of last century [2].

The surgical management of breast cancer has significantly evolved since the original mastectomy technique devised by Halstead in the 1890s [3]. Increasing use of neoadjuvant chemotherapy and developments in oncoplastic techniques have led to a rise in breast-conserving surgery with greater preservation of subcutaneous fat after removal of breast parenchyma [4]. Reconstruction is now integral to the surgical management of breast cancer leading to reduced psychosocial morbidity and greater patient satisfaction [5]. Following the development of silicone implants in the 1960s by Cronin and Gerow, implant-based breast reconstruction (IBBR) gained popularity [6]. Immediate IBBR is currently the most prevalent reconstructive procedure performed in the UK [7].

Initial IBBRs took place in the pre-pectoral plane but were replaced by subpectoral techniques in the 1970s because of increased rates of capsular contracture, skin flap necrosis, infection and implant exposure and poor aesthetic results [5]. Despite the benefits of muscular coverage leading to reduced implant exposure and improved cosmesis, subpectoral reconstruction has been associated with increased postoperative pain, animation deformity and functional deficits [8].

Pre-pectoral IBBR has gained renewed interest among surgeons [4, 5]. This is in part due to evolving techniques in oncoplastic surgery such as operating in the mastectomy plane to preserve thicker skin flaps in skin-sparing and nipple-sparing mastectomies [9]. Furthermore, new technologies help reduce incidence of complications associated with pre-pectoral IBBR. For example, indocyanide green predicts occurrence of skin flap necrosis intra-operatively thus allowing adaptation of operative technique if required [9].

Its rise in popularity has also been attributed to the use of Acellular Dermal Matrices (ADM) which are widely used by surgeons for both pre-pectoral and sub-pectoral reconstructions [10]. Despite this, evidence supporting the benefits of ADM in IBBR is still debated. A randomized clinical trial by Lohmander et al. demonstrated that ADM did not significantly reduce postoperative complications in sub-pectoral IBBR, raising questions about its routine use in implant-based breast reconstruction [11]. While this study is specific to sub-pectoral reconstruction, its findings underscore the need for further investigation into the safety and efficacy of ADM in reconstructive settings, particularly in pre-pectoral IBBR, where large-scale evidence remains limited.

In pre-pectoral IBBR, ADM is said to improve cosmetic appearance and provide more flexibility with reconstructive size [12]. Nonetheless, it has also been reported to increase the risk of infection, seroma and skin necrosis [13]. The United States Food and Drug Administration (FDA) issued a statement in 2021 highlighting risks associated with the use of ADM and re-iterating that the FDA has not approved or cleared ADM for use in IBBR [14]. Further safety concerns have been raised recently with Surgimend, an ADM produced by Integra, being recalled due to higher levels of endotoxin in the product causing post-operative fever [15].

Current evidence in literature regarding pre-pectoral ADM use for IBBR is limited and there is scant comparison with non-ADM use in the same setting. Reporting of complications and patient quality of life is inconsistent. A systematic review was performed to explore surgical outcomes and quality of life for patients undergoing pre-pectoral IBBR with or without ADM.

## Methodology

This review was registered with the International prospective register of systematic reviews (PROSPERO), part of the National Institute for Health Research (NIHR). Registration is as follows: PROSPERO 2023 CRD42023389072 [16].

### Study question

This study aims to investigate surgical outcomes for patients undergoing pre-pectoral IBBR with or without ADM, defined by post-operative complications, implant failure (defined by loss of implant) and patient reported quality of life.

### Literature search

A systematic literature search was conducted with the assistance of the Royal College of Surgeons of England. Databases searched were Ovid Medline, Embase and Cochrane Central Register of Controlled Trials (CENTRAL) and Cochrane Database of Systematic Reviews (CDSR). The search timeframe was 10 years and included the following terms in various combinations and forms:

- Acellular dermal matrix (ADM).
- Mammaplasty, breast implantation, breast reconstruction.
- Mastectomy.
- Breast cancer.
- Post-operative complications, treatment outcomes.
- Quality of life.

A total of 147 studies were identified after removal of 81 duplicates. References of included studies were also screened for inclusion suitability. Additionally, eligible papers suggested by reviewers and not included in the initial search string were included.

### Study selection and data extraction

**Fig. 1:**
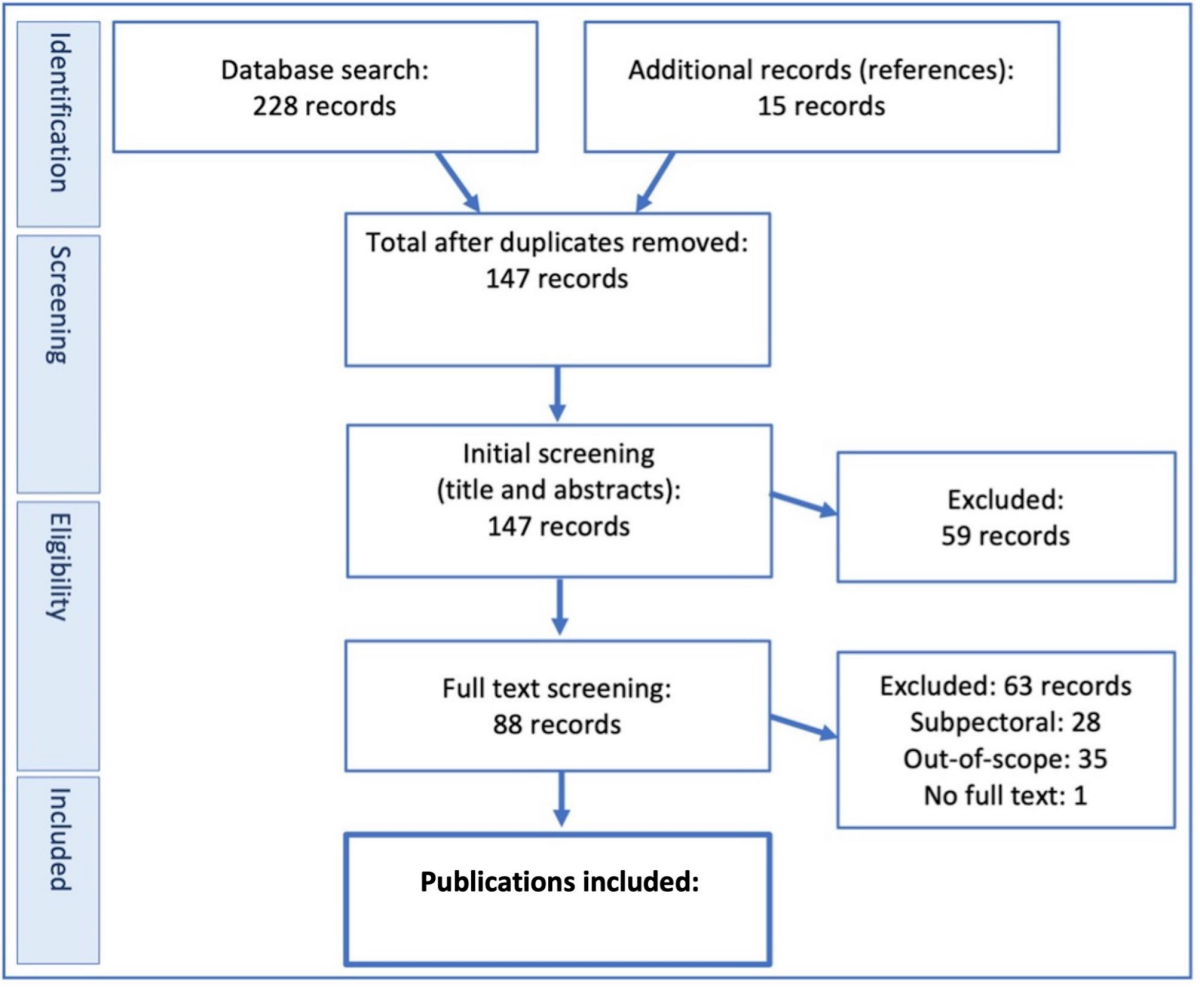
PRISMA chart for publication selection [17]

Studies were independently evaluated according to PICO criteria (Table 1), inclusion criteria (Table 2) and exclusion criteria (Table 3) by two review team members. Titles and abstracts were initially screened using exclusion criteria and then read in full (Fig. 1) [17]. A minimum follow-up time of greater than 12 months was stipulated for papers that investigated non-immediate complications, such as capsular contracture and rippling. For papers investigating only immediate complications, a follow-up time of at least 4 weeks was accepted.

This study focused solely on reconstructions with and without ADM. Despite growing interest in synthetic meshes for implant-based breast reconstruction, studies involving mesh were deliberately excluded from this study. This decision was made to avoid broadening the review’s scope and introducing additional heterogeneity, which could compromise the internal reliability of results. Future studies may also be needed to investigate outcomes associated with synthetic meshes in IBBR.

Conflict on difference of opinion between reviewers was resolved in discussion with senior authors. Data from studies that passed initial and full screening were extracted by two reviewers and cross-checked. Data were collected in a standardized spreadsheet according to categories (patient demographics and outcome measures). With regards to patient quality of life, all patient reported outcome measure scores mentioned in studies were included in the initial phase, for analysis later.

**Table 1:** Study population, intervention, comparison and outcomes (PICO)

Patient	1) Females undergoing pre-pectoral implant-based breast reconstruction with or without ADM 2) women undergoing reconstruction for cancer treatment or prophylaxis 3) immediate or delayed reconstruction 4) unilateral or bilateral reconstruction
Intervention	use of acellular dermal matrices (ADM) during breast reconstruction procedures
Comparison	non-use of ADM during breast reconstruction procedures
Outcome	operative success, defined by: 1) complications 2) failure (loss of implant) 3) patient quality of life

**Table 2:** Inclusion criteria

Inclusion Criteria	- Follow-up of at least 12 months in studies examining all complications - Follow-up of at least 28 days in studies examining only immediate complications - Must fulfil criteria as defined by PICO

**Table 3:** Exclusion criteria

Exclusion Criteria	- secondary reconstructive procedures such as reconstruction revision - aesthetic or cosmetic procedures - sub-pectoral implant placement - non-implant-based reconstruction, for example, autologous free flaps - non-English language - animal or cadaveric studies - systematic review including papers already present in results

### Study quality

Study quality and risk of bias was assessed using the Newcastle-Ottawa scale. Studies were independently reviewed by two team members and scores were correlated.

### Statistical analysis

The statistical analyses were performed on Microsoft Excel and R (version 4.0.3) software [18]. Provided data for each complication across studies were combined to calculate complication rates and risk ratios (RR) with 95% confidence intervals (CIs). Not all studies mentioned every complication; data for rate calculations were only taken from studies that included a certain complication. For two-arm studies, forest plots were created comparing the ADM and non-ADM groups for each complication. A random effects model was fitted; heterogeneity and *p*-value for overall effect are presented on forest plots (appendix 1), with RR, 95% CIs, and weight. A *p*-value of less than 0.05 was considered statistically significant.

## Results

A total of 22 publications were eligible for analysis after application of inclusion and exclusion criteria. This included 3072 patients and 3822 breasts (2898 with ADM-incorporated reconstructions, 924 without ADM-incorporated reconstructions).

### Study and operative characteristics

The majority (*n* = 20, 87%) of included studies were retrospective in nature; 2 included studies were prospective. 4 studies were two-arm and comparative [19–22]. Other studies reported ADM use alone ( [23–32] and non-ADM use alone) [10, 33, 34]. 6 papers compared pre-pectoral to sub-pectoral IBBR, in these data pertaining solely to pre-pectoral reconstruction was extracted [13, 35–39].

14 studies reported solely immediate direct-to-implant (DTI) reconstructions, 4 reported immediate tissue expander (TE) reconstruction and the remaining 4 reported a mixture of one and two stage reconstructions. Overall, 2725 out of 2898 ADM breast reconstructions (93%) and 531 out of 924 non-ADM breast reconstructions (57.4%) were direct-to-implant. For the ADM cohort, 93 breasts were reconstructed using tissue expanders (3.2%), compared to 377 (40.8%) of breasts reconstructed without ADM. 1.1% (*n* = 30) of ADM breast reconstructions and 2% (*n* = 20) of non-ADM reconstructions were delayed.

With regards to the placement of ADM (when used), 8 studies (36%) described anterior coverage and 7 studies (32%) described complete wrapping of implant or tissue expander with ADM. Unfortunately, due to not all studies documenting ADM coverage methods in reconstruction, sub-group analysis could not be performed to assess whether this affected operative outcomes.

### Study quality

**Fig. 2:**
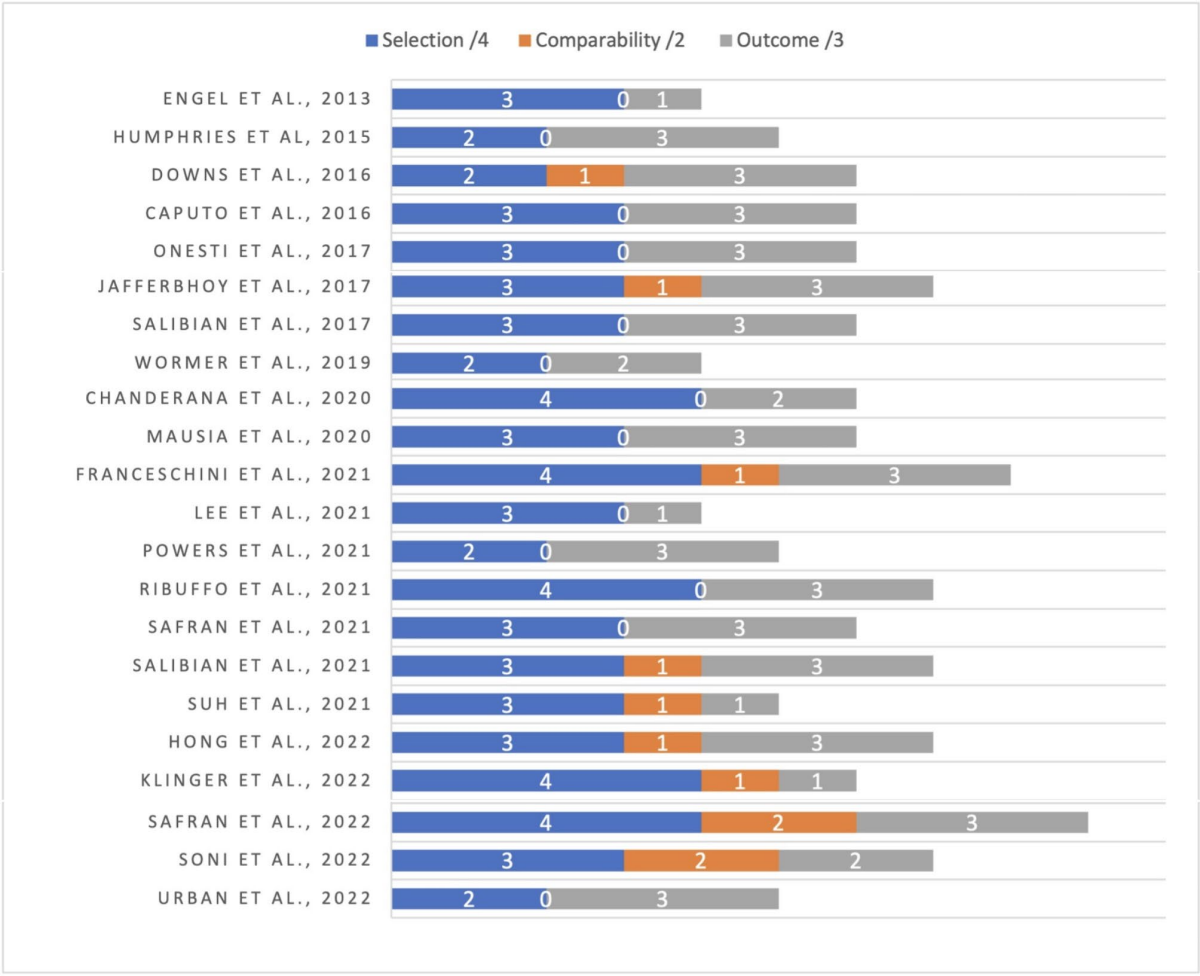
Newcastle Ottawa score – study quality and risk of bias assessment

Study quality and risk of bias were assessed for selection, comparability and outcome using the Newcastle-Ottawa scale (Fig. 2). The average score was 6 out of 9. Generally, studies lost points for comparability and selection of representative groups – likely due to being largely retrospective in nature and frequently single-surgeon studies. Safran et al., (2022) [20] and Franceschini et al., [13] were scored highest – 9 and 8, respectively. Lee et al., [38], Engel et al., [34] and Wormer et al., [39] scored 4 due to poor comparability and inadequate documentation of follow-up.

Follow-up was reported as mean (with standard deviation) or median (with range). 5 studies had follow-up times of less than 1 year; only 6 studies had follow-up times of more than 2 years. Klinger et al., 2022 [19] reported assessing pain and aesthetic outcomes at 12 months but did not provide a timeframe for assessment of other complications.

### Patient characteristics

**Table 4:** Patient demographics and factors in included studies

	Number of Studies	Number of Patients	ADM		No ADM		*p*-value
			Mean	SD	Mean	SD	
Age	20	2,886	52.7	3.0	47.5	3.7	0.522
BMI	19	2,859	24.7	2.3	24.9	2.1	0.269
Smoking status (%)	16	2,819	8.5	6.5	5.7	6.6	0.567
Diabetes (%)	11	2,382	2.5	2.6	5.3	2.5	0.367
Neo-adjuvant chemotherapy (%)	16	2,794	16.4	12.7	30.9	12.0	0.104
Adjuvant chemotherapy (%)	8	2,065	19.7	20.3	11.7	13.0	0.347
Neo-adjuvant radiotherapy (%)	15	2,571	5.9	22.3	7.3	6.5	0.492
Adjuvant radiotherapy (%)	9	2,155	78.8	9.4	19.1	5.6	0.091
Skin sparing mastectomy (%)	7	1,743	58.0	31.7	29.6	44.7	0.703
Nipple sparing mastectomy (%)	4	1,640	44.6	27.5	62.7	7.4	0.898

Demographic and surgical data were extracted and compared between ADM and non-ADM breast reconstructions. The average age was 52.7 for ADM reconstructions and 47.5 for non-ADM reconstructions. There were no statistically significant differences observed between patient characteristics (Table 4), however not all papers provided data for these measurements. Notably, Salibian et al., 2017 [33] and Humphries et al., 2015 [26] did not include any data regarding population age, BMI or prevalence of diabetes. Caputo et al., 2016 [27] did not comment on BMI, smoking or diabetes in their patient characteristics.

### Complications and failure

**Table 5:** Comparison of complications for ADM use versus non-ADM use including risk ratio (RR), 95% confidence intervals (CI), and *p*-value

Complication	Number of papers		Number of Complications		Rate (%)		Risk Ratio (95% CI)	*p*-value
	ADM	no ADM	ADM	no ADM	ADM	no ADM		
Overall Complications	8	5	565	89	22.0%	18.9%	1.16 (0.95–1.42)	0.140
Seroma	12	6	197	33	8.0%	6.8%	1.17 (0.82–1.67)	0.380
Haematoma	10	6	58	9	2.4%	1.9%	1.30 (0.65–2.61)	0.456
Minor Infection	15	4	164	16	6.3%	8.9%	0.69 (0.42–1.12)	0.132
Wound Dehiscence	9	5	96	27	3.7%	6.6%	0.61 (0.40–0.92)	**0.019**
Flap/NAC Necrosis	10	7	93	32	4.2%	4.1%	1.04 (0.71–1.55)	0.822
Capsular Contracture	7	5	74	67	3.0%	9.8%	0.31 (0.22–0.42)	**< 0.0001**
Implant Removal	12	7	152	49	7.1%	6.7%	1.07 (0.78–1.46)	0.670
Rippling	6	5	76	55	4.2%	8.2%	0.50 (0.36–0.71)	**< 0.0001**
Rotation	3	3	5	2	0.3%	0.4%	0.55 (0.11–2.83)	0.475

Complications are shown in Table 5. Overall complication rate was defined as presence of at least one complication per reconstruction. Incidence of overall complication was 22.0% in the ADM group versus 18.9% in the non-ADM group. However, this parameter was only measured in 9 studies (4 two-arm); other studies did not comment on the distribution of complications amongst reconstructions. The complication with the greatest total incidence was seroma. The most mentioned complications across all studies were minor infection and implant removal (16 studies each). Complications with significant difference observed between the ADM and non-ADM groups were wound dehiscence, capsular contracture and rippling; with a decreased risk being observed in the ADM group. Among these complications, the risk ratios were 0.61, 0.31 and 0.55 respectively.

### Two-arm studies

Forest plots for each complication assessing heterogeneity are available in appendix 1. Given that only 4 studies were two-arm, heterogeneity was high across all complications and in some instances underpowered.

### Subgroup analysis

Subgroup analysis was performed for studies comparing DTI and TE based immediate reconstruction for all complication outcomes except rotation, as this was reported in studies where reconstruction was solely DTI. The subgroup effect was not statistically significant for any outcome. As only 3 studies reported delayed reconstruction, subgroup analysis was not performed with this cohort [26, 34, 35].

Subgroup analysis was also performed for ADM use and non-ADM use for patient characteristics: age, BMI and smoking status. There was a weak correlation observed between these factors and each complication, demonstrating no overall subgroup effect.

### Patient quality of life

A total of 6 studies reported some form of patient reported outcome measure; 2 used BREAST-Q (including satisfaction with breast, operative outcome, psychosocial and sexual wellbeing) [22, 23], 1 used EORT-QLQ and QLQ-BR23 (European Organisation for research and treatment of cancer questionnaire for all cancer and breast cancer patients, respectively) [30], 1 used Hospital Anxiety and Depression Score [38] and 2 used self-devised patient questionnaires [13, 34].

Safran et al., 2021 [22] were the only study to perform BREAST-Q scores on patients receiving both ADM and non-ADM reconstructions; scores were collected at 6 months and 1 year postoperatively. Only overall satisfaction was reported – 54% for ADM and 55% for non-ADM reconstructions. Hong et al. (ADM use) reported consistently high scores in all parameters (> 80%) [23]. Among ADM patients, body image was 80% (with higher score representing a greater health outcome), sexual wellbeing 65–73%, emotional health 86% and overall life and quality of health score 77%.

## Discussion

With the renaissance of pre-pectoral IBBR, it is important that surgeons have an adequate evidence base to enable operative planning in the patient’s best interest.

Arguably, pre-pectoral IBBR is beneficial both in the short term (reduced operative time and postoperative pain) and the long term (reduced risk of animation deformity and functional loss) [8]. The use of ADM has significantly contributed to increased pre-pectoral IBBR rates, alongside implant technology improvement and access to intra-operative perfusion assessment [4, 5]. Studies have suggested that ADM use improves aesthetic outcomes via improved implant coverage and additional tissue support [40], however claims of these benefits are not substantiated in randomised clinical trials and are often based on author opinion alone [41].

It has been suggested that the benefits associated with ADM in pre-pectoral IBBR can be replicated with careful patient selection, operative planning and technique. Comparable aesthetic outcomes and complication rates were demonstrated in a retrospective, single-surgeon study between ADM and non-ADM pre-pectoral IBBR [42]. ADM is also costly – the same study estimated the institutional cost saving would be $3 million – $6 million (unilateral – bilateral cases) if ADM were no longer used [42].

This review has demonstrated no significant difference between complication and failure rates between ADM and non-ADM pre-pectoral IBBR for seroma/haematoma, infection, skin/NAC necrosis and implant rotation. Overall complications (as defined by presence of at least 1 complication per reconstruction) was slightly higher in the ADM group but not significantly so. This parameter was also only included in 9 studies. There is no significant difference in implant failure or explantation between groups. This is consistent with results from Salibian and Safran [21, 22]. Capsular contracture, wound dehiscence and implant rippling had significant differences between groups however results demonstrated a high rate of heterogeneity and were from relatively small sample sizes, thus caution should be applied when generalising this finding to the wider population.

Further research has been conducted on the use of ADM in sub-pectoral IBBR, which is out of the scope of this review, but nonetheless of note. Lohmander et al. conducted a randomized clinical trial comparing ADM and non-ADM use in sub-pectoral IBBR and found no significant difference in key post-operative complications [11]. Their findings suggest limited benefit to ADM use in sub-pectoral settings. Furthermore, recent clinical recommendations from the Group for Reconstructive and Therapeutic Advancements (GReTA) advise sub-pectoral IBBRs with or without ADM, citing a very low certainty of evidence for the important outcomes associated with such reconstructions [43]. The panel felt there was a strong need for better evidence on the topic. Whilst in line with the conclusions of this review, the anatomical and procedural differences between sub-pectoral and pre-pectoral reconstruction must be considered before extrapolating findings. The absence of direct evidence for ADM use in pre-pectoral IBBR highlights the need for further research in this area.

This study has also highlighted a lack of external validity of existing evidence in the literature on this topic. Limitations include heterogeneity of data (for example the high number of TEs in the non-ADM cohort) and lack of available evidence directly comparing ADM and non-ADM use in pre-pectoral IBBR in a single study setting. Meta-analysis was limited by studies being mainly retrospective and the scarcity of randomised data. The disparity in sample sizes between ADM and non-ADM groups may impact the reliability of the results and represents a risk of bias. Potential confounding factors such as the degree of ADM coverage around implants were not ascertainable from the literature. Severity and grade of capsular contracture was recorded variably among studies – with some documenting all incidences (ungraded) and others documenting only Baker Grade 3 and above. This reduced reliability of extracted data for comparison.

The lack of externally valid evidence and significant limitations of data on this topic is significant given the high frequency of pre-pectoral IBBR procedures performed in the UK and worldwide, and the costs associated with ADM use. Further prospective, randomised and large-scale studies would provide robust evidence for clinicians, with data captured from more homogenous patient groups. However, given the challenges associated with recruiting participants for randomised trials, multi-centre observational, cohort, or well-designed case-control studies may provide valuable insights. International collaboration may help to overcome the challenges of regional variability and recruitment. Further research into patient reported outcome measures in these populations is also required.

## Conclusion

In conclusion, there is no correlation between ADM use in pre-pectoral IBBR and decreased complications except for capsular contracture, wound dehiscence and rippling, and these results should be interpreted with caution given significant heterogeneity and small sample sizes.

Lack of significant difference between use of ADM versus non-use has significant implications for ongoing clinical practice and healthcare legislature in breast cancer reconstruction given the widespread use of ADM and associated cost. However, limited data exists on this topic and further evidence is required to ascertain surgical outcomes accurately and reliably. Future research should explore study methodologies including large-scale, randomised studies and multi-centre observational or cohort studies.

## Electronic supplementary material

Below is the link to the electronic supplementary material.

Supplementary Information 1

## Data Availability

Data is provided within the manuscript or supplementary information files.
