# Supplementary material for: Does the use of Acellular Dermal Matrices (ADM) in women undergoing pre-pectoral implant-based breast reconstruction increase operative success versus non-use of ADM in the same setting? A systematic review
Source: BMC Cancer. 2024 Sep 27;24:1186. doi: 10.1186/s12885-024-12978-0 (PMC11437634; doi:10.1186/s12885-024-12978-0)

Total Complications

Study	ADM Events	ADM Total	No ADM Events	No ADM Total	RR	95%-CI	Weight
Safran, 2022	58	252	1	82	12.83	[2.55; 64.49]	25.8%
Salibian, 2021	9	35	7	41	1.50	[0.62; 3.62]	35.6%
Safran, 2021	22	77	11	44	1.14	[0.61; 2.13]	38.6%
**Random effects model**		**364**		**167**	**2.35**	**[0.63; 8.72]**	**100.0%**

Heterogeneity: I^2 = 73%, τ^2 = 1.0572, p = 0.02
Test for overall effect: z = 1.28 (p = 0.20)

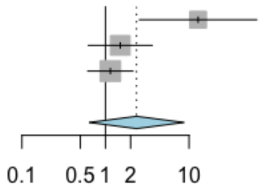

Minor Infection

Study	ADM Events	ADM Total	No ADM Events	No ADM Total	RR	95%-CI	Weight
Klinger, 2022	0	14	1	16	0.38	[0.02; 8.54]	29.3%
Salibian, 2021	1	35	2	41	0.59	[0.06; 6.17]	38.0%
Safran, 2021	15	77	0	44	17.82	[1.09; 290.63]	32.7%
**Random effects model**		**126**		**101**	**1.58**	**[0.15; 16.38]**	**100.0%**

Heterogeneity: I^2 = 54%, τ^2 = 2.3218, p = 0.11
Test for overall effect: z = 0.38 (p = 0.70)

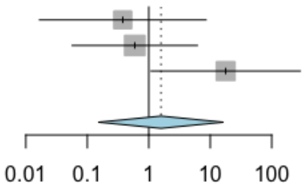

Seroma

Study	ADM Events	ADM Total	No ADM Events	No ADM Total	RR	95%-CI	Weight
Klinger, 2022	0	14	4	16	0.13	[0.01; 2.15]	23.4%
Salibian, 2021	1	35	3	41	0.40	[0.04; 3.61]	38.6%
Safran, 2021	3	77	1	44	1.70	[0.18; 15.65]	38.0%
**Random effects model**		**126**		**101**	**0.53**	**[0.13; 2.09]**	**100.0%**

Heterogeneity: I^2 = 5%, τ^2 = 0.0092, p = 0.35
Test for overall effect: z = -0.91 (p = 0.36)

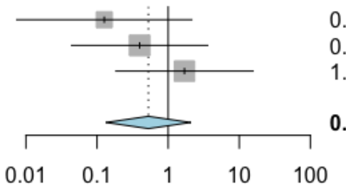

Wound Dehiscence

Study	ADM Events	ADM Total	No ADM Events	No ADM Total	RR	95%-CI	Weight
Klinger, 2022	1	14	0	16	3.37	[0.15; 76.85]	21.9%
Salibian, 2021	2	35	2	41	1.16	[0.17; 7.83]	58.9%
Safran, 2021	0	77	1	44	0.27	[0.01; 7.53]	19.2%
**Random effects model**		**126**		**101**	**1.11**	**[0.26; 4.78]**	**100.0%**

Heterogeneity: I^2 = 0%, τ^2 = 0, p = 0.55
Test for overall effect: z = 0.14 (p = 0.89)

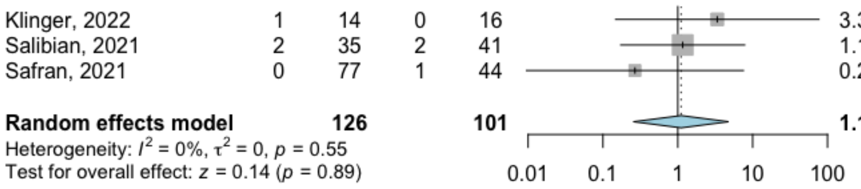

Haematoma

Study	ADM Events	ADM Total	No ADM Events	No ADM Total	RR	95%-CI	Weight
Klinger, 2022	0	14	1	16	0.38	[0.02; 8.54]	32.3%
Salibian, 2021	1	35	0	41	3.54	[0.15; 83.92]	31.4%
Safran, 2021	3	77	0	44	4.02	[0.21; 76.10]	36.4%
**Random effects model**		**126**		**101**	**1.80**	**[0.31; 10.60]**	**100.0%**

Heterogeneity: I^2 = 0%, τ^2 = 0, p = 0.49
Test for overall effect: z = 0.65 (p = 0.52)

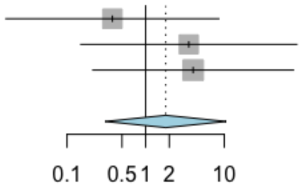

Flap / Nipple Necrosis

Study	ADM Events	ADM Total	No ADM Events	No ADM Total	RR	95%-CI	Weight
Klinger, 2022	0	14	0	16			0.0%
Salibian, 2021	1	35	4	41	0.30	[0.04; 2.52]	24.8%
Safran, 2021	3	77	8	44	0.24	[0.07; 0.80]	75.2%
**Random effects model**		**126**		**101**	**0.25**	**[0.09; 0.73]**	**100.0%**

Heterogeneity: I^2 = 0%, τ^2 = 0, p = 0.85
Test for overall effect: z = -2.55 (p = 0.01)

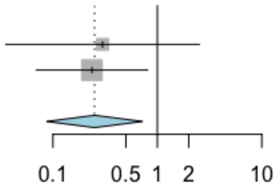

Capsular Contraction

Study	ADM Events	ADM Total	No ADM Events	No ADM Total	RR	95%-CI	Weight
Safran, 2022	17	252	1	82	5.75	[0.77; 43.16]	57.3%
Salibian, 2021	0	35	2	41	0.27	[0.01; 5.74]	42.7%
**Random effects model**		**287**		**123**	**1.57**	**[0.08; 30.03]**	**100.0%**

Heterogeneity: I^2 = 63%, τ^2 = 2.9058, p = 0.10
Test for overall effect: z = 0.30 (p = 0.77)

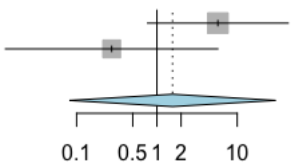

Rippling

Study	ADM Events	ADM Total	No ADM Events	No ADM Total	RR	95%-CI	Weight
Safran, 2022	17	252	1	82	7.67	[0.76; 77.71]	30.0%
Klinger, 2022	1	14	5	16	0.23	[0.03; 1.73]	32.8%
Salibian, 2021	2	35	7	41	0.31	[0.06; 1.56]	37.2%
**Random effects model**		**301**		**139**	**0.73**	**[0.09; 5.85]**	**100.0%**

Heterogeneity: I^2 = 68%, τ^2 = 2.3419, p = 0.05
Test for overall effect: z = -0.29 (p = 0.77)

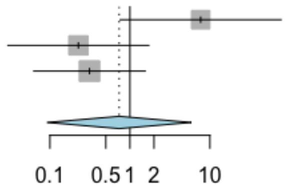

Removal

Study	ADM Events	ADM Total	No ADM Events	No ADM Total	RR	95%-CI	Weight
Klinger, 2022	0	14	0	16			0.0%
Salibian, 2021	4	35	5	41	0.93	[0.27; 3.22]	73.9%
Safran, 2021	5	77	0	44	6.32	[0.36; 111.65]	26.1%
**Random effects model**		**126**		**101**	**1.54**	**[0.30; 7.97]**	**100.0%**

Heterogeneity: I^2 = 30%, τ^2 = 0.5556, p = 0.23
Test for overall effect: z = 0.51 (p = 0.61)

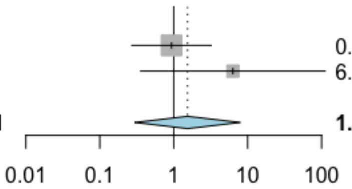

Rotation

Study	ADM Events	ADM Total	No ADM Events	No ADM Total	RR	95%-CI	Weight
Safran, 2022	2	252	0	82	1.00	[0.04; 24.71]	100.0%
Klinger, 2022	0	14	0	16			0.0%
**Random effects model**		**266**		**98**	**1.00**	**[0.04; 24.71]**	**100.0%**

Heterogeneity: I^2 = NA%, τ^2 = NA, p = NA
Test for overall effect: z = 0.00 (p = 1.00)

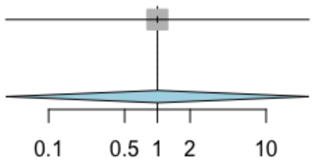

Supplement: Supplementary file 1 — Supplementary Information 1 [file 12885_2024_12978_MOESM1_ESM.pdf]
